# Effects of Conserved Wedge Domain Residues on DNA Binding Activity of *Deinococcus radiodurans* RecG Helicase

**DOI:** 10.3389/fgene.2021.634615

**Published:** 2021-02-04

**Authors:** Sun-Wook Jeong, Min-Kyu Kim, Lei Zhao, Seul-Ki Yang, Jong-Hyun Jung, Heon-Man Lim, Sangyong Lim

**Affiliations:** ^1^Radiation Research Division, Korea Atomic Energy Research Institute, Jeongeup, South Korea; ^2^Department of Biological Sciences, College of Biological Sciences and Biotechnology, Chungnam National University, Daejeon, South Korea; ^3^Department of Radiation Science and Technology, University of Science and Technology, Daejeon, South Korea

**Keywords:** *Deinococcus radiodurans*, radiation resistance, DNA repair, RecG helicase, wedge domain

## Abstract

*Deinococcus radiodurans* is extremely resistant to ionizing radiation and has an exceptional ability to repair DNA damage caused by various DNA-damaging agents. *D. radiodurans* uses the same DNA-repair strategies as other prokaryotes, but certain proteins involved in the classical DNA repair machinery have characteristics different from their counterparts. RecG helicase, which unwinds a variety of branched DNA molecules, such as Holliday junctions (HJ) and D-loops, plays important roles in DNA repair, recombination, and replication. Primary sequence analysis of RecG from a number of bacterial species revealed that three amino acids (QPW) in the DNA-binding wedge domain (WD) are well-conserved across the *Deinococcus* RecG proteins. Interactions involving these conserved residues and DNA substrates were predicted in modeled domain structures of *D. radiodurans* RecG (DrRecG). Compared to the WD of *Escherichia coli* RecG protein (EcRecG) containing FSA amino acids corresponding to QPW in DrRecG, the HJ binding activity of DrRecG-WD was higher than that of EcRecG-WD. Reciprocal substitution of FSA and QPW increased and decreased the HJ binding activity of the mutant WDs, EcRecG-WD_QPW_, and DrRecG-WD_FSA_, respectively. Following *γ*-irradiation treatment, the reduced survival rate of DrRecG mutants (Δ*recG*) was fully restored by the expression of DrRecG, but not by that of EcRecG. EcRecG_QPW_ also enhanced *γ*-radioresistance of Δ*recG*, whereas DrRecG_FSA_ did not. Δ*recG* cells complemented *in trans* by DrRecG and EcRecG_QPW_ reconstituted an intact genome within 3 h post-irradiation, as did the wild-type strain, but Δ*recG* with EcRecG and DrRecG_FSA_ exhibited a delay in assembly of chromosomal fragments induced by *γ*-irradiation. These results suggested that the QPW residues facilitate the association of DrRecG with DNA junctions, thereby enhancing the DNA repair efficiency of DrRecG.

## Introduction

*Deinococcus radiodurans* is well known for its extreme resistance to lethal doses of ionizing radiation (IR) and many other DNA damaging agents, including mitomycin C (MMC), UV-C radiation, and desiccation (Slade and Radman, 2011). This remarkable resistance is thought to be attributed to its highly efficient DNA repair capacity and various anti-oxidative systems (Lim et al., 2019). In *D. radiodurans*, extensive IR-induced DNA double-strand breaks (DSBs), which are the most lethal form of DNA damage, can be mended within a few hours (Zahradka et al., 2006). The rapid reconstruction of an intact genome from hundreds of chromosomal fragments is achieved through extended synthesis-dependent strand annealing (ESDSA), followed by homologous recombination (HR; Zahradka et al., 2006).

ATP-dependent duplex DNA unwinding enzymes, termed DNA helicases, are prevalent in all kingdoms of life and play important roles in the processes of DNA replication, repair, recombination, etc. (Brosh and Matson, 2020). The human genome encodes for 31 nonredundant DNA helicases (Umate et al., 2011). Given their fundamental roles in DNA metabolism, mutations in some of these genes are associated with certain human diseases characterized by premature aging and cancer, including Xeroderma Pigmentosum, Cockayne Syndrome, and Werner Syndrome (Uchiumi et al., 2015). Since DNA helicase was first discovered in the model bacterium *Escherichia coli* (Brosh and Matson, 2020), the *E. coli* helicases have been intensively studied, and their function has been compared to that of counterparts identified in different organisms. In *E. coli*, HR initiation follows the RecBCD pathway. The RecBCD complex binds to double-stranded DNA (dsDNA) ends and unwinds and degrades the DNA by using a combination of helicase and nuclease activities, which promotes the repair of DSB (Rocha et al., 2005). *D. radiodurans* is devoid of RecB and RecC proteins but possesses a RecD homolog named RecD2 (Wang and Julin, 2004). Since RecD2 is present in *recBC*-minus organisms, it is not associated with RecBC (Montague et al., 2009). The *D. radiodurans* RecD2 protein is a DNA helicase with 5'-3' polarity and low processivity (Wang and Julin, 2004). *recD2* mutants are more sensitive than wild type cells to the cytotoxic effect of *γ*-irradiation, UV light, and hydrogen peroxide (H_2_O_2_; Servinsky and Julin, 2007; Zhou et al., 2007), but *recD2* mutations does not alter the sensitivity of *D. radiodurans* to treatment with mitomycin C (MMC), methyl methanesulfonate, and hydroxyurea (Servinsky and Julin, 2007; Montague et al., 2009). *D. radiodurans* lacks not only RecBC, but also exonuclease I (SbcB), hence DSBs in *D. radiodurans* are repaired by the RecFOR pathway, which is significantly more common than RecBCD in bacterial genomes (Rocha et al., 2005). RecFOR-dependent DSB repair is initiated by unwinding duplex DNA, followed by DNA end resection that degrades the broken ends in the 5'–3' direction to obtain 3' single-stranded DNA (ssDNA) tails (Rocha et al., 2005).

In *E. coli*, RecQ is the major helicase implicated in the RecFOR pathway and in nucleolytic degradation catalyzed by RecJ; however, in *D. radiodurans*, RecQ is dispensable (Bentchikou et al., 2010). Different results have been reported with respect to *recQ* mutant phenotypes: a *recQ* mutant strain was reported to be sensitive to γ-irradiation, UV, H_2_O_2_, and MMC (Huang et al., 2007), whereas another study showed that *recQ* mutant cells display wild-type resistance to γ-irradiation (Bentchikou et al., 2010). Recently, RecD2 and RecQ proteins from *D. radiodurans* were reported to be able to unwind guanine quadruplex (G4) DNA structures (Khairnar et al., 2019; Xue et al., 2020). Instead of RecQ, in *D. radiodurans*, the UvrD helicase, which can unwind duplex DNA in both the 3'–5' and 5'–3' directions (Stelter et al., 2013), plays a critical role in DSB repair and reconstitution of the genome following IR exposure (Bentchikou et al., 2010). However, the *uvrD* mutant still retained significant radio-resistance as compared to a repair-deficient *recA* mutant strain, suggesting that the redundant activity of other helicase(s) is responsible for the residual DNA repair capacity observed (Bentchikou et al., 2010).

The RecFOR complex loads RecA onto ssDNA substrates, thereby resulting in the formation of a RecA nucleoprotein filament that searches for homology and then invades the double-stranded homologous DNA (Bentchikou et al., 2010). RecA-mediated strand invasion creates a D-loop, and primes DNA polymerase III (Pol III)- and/or Pol I-dependent DNA synthesis (Slade et al., 2009). DNA synthesis proceeds *via* a migrating D-loop, in which the unwinding of the dsDNA template may be mediated by UvrD, RecD2, RecQ, RuvAB, and/or other helicases (Slade and Radman, 2011). The RuvABC system can displace and resolve a four-way DNA intermediate named the Holliday junction (HJ). The RuvAB and RuvC proteins catalyze branch migration and the resolution of HJ recombination intermediates, respectively (Rocha et al., 2005). The *D. radiodurans ruvB* mutant is modestly sensitive to UV light, *γ*-irradiation, and MMC (Kitayama et al., 1997). However, the fact that inactivation of both *ruv* and *recG* resulted in a more dramatic increase in the sensitivity of cells to DNA damaging-agents has led to the suggestion that RecG and RuvABC are part of two overlapping pathways for processing intermediates in HR and DNA repair (Lloyd and Rudolph, 2016). The deletion of *recG* resulted in a growth delay and a decrease in the resistance of *D. radiodurans* to γ-irradiation and H_2_O_2_ (Wu et al., 2009).

RecG, a monomeric dsDNA translocase that unwinds a variety of branched DNA molecules, such as replication forks, HJs, D-, and R-loops (Lloyd and Rudolph, 2016), consists of an N-terminal wedge domain (WD), two RecA-like helicase domains, and a C-terminal translocation in RecG (TRG) motif (Fairman-Williams et al., 2010). The WD, which is not found in other DNA helicases, provides specificity for binding branched DNA structures (Rudolph et al., 2010). Amino acid sequence analysis of WDs of RecGs from *Deinococcus* species revealed that a “Gln(Q)-Pro(P)-Trp(W)” residue motif is highly conserved in *Deinococus* RecG proteins. In this study, we found that QPW residues contributed to strong binding of RecG to HJ and, consequently, enhanced the ability of RecG to repair DNA damage in *D. radiodurans*.

## Materials and Methods

### Bacterial Strains and Culture Conditions

*Deinococus radiodurans* R1 (ATCC13939) and its isogenic *recG* mutant strains (Δ*recG*), which had been previously constructed (Jeong et al., 2016), were cultivated at 30°C in TGY broth (0.5% tryptone, 0.1% glucose, and 0.3% yeast extract) with aeration or on TGY plates supplemented with 1.5% Bacto-agar. The *E. coli* strain DH5α was used for routine cloning experiments. *Escherichia coli* strains were grown at 37°C in Luria-Bertani (LB) medium or on LB plates solidified with 1.5% Bacto-agar. Antibiotics were added to the medium if necessary: ampicillin, 100 μg/ml (*E. coli*), and chloramphenicol, 3 μg/ml (*D. radiodurans*).

### Plasmid Construction

The pRADZ3 shuttle vector, which functions in *E. coli* as well as in *D. radiodurans*, contains the *groEL* promoter for constitutive gene expression (Jeong et al., 2016). Complete *recG* coding sequences were PCR-amplified from genomic DNA of *D. radiodurans* R1 and *E. coli* MG1655 by using DR1916F/R and B3652F/R primer pairs, respectively, carrying the SpeI and BamHI restriction sites. The PCR products were cloned into pRADZ3 at the *Spe*I and *Bam*HI sites to generate the plasmids pDrRecG and pEcRecG, respectively. These plasmids were transformed into Δ*recG* for complementation studies. For transformation, *D. radiodurans* cells from exponentially growing cultures were collected by centrifugation and concentrated 50-fold in TGY supplemented with 30 mM CaCl_2_. The cell mixture (100 μl) containing the constructed plasmid DNAs was held on ice for 30 min and then incubated at 32°C for 90 min. The transformation mixture was diluted 10-fold with TGY broth and incubated at 30°C for 5 h with aeration, prior to being plated on drug-selective agar. The partial nucleotide sequence of *recG* encoding DrRecG lacking the N-terminal region (residues 1–99) was PCR-amplified using DR1916-*Δ*NF/-ΔNR primer pairs and cloned into pRADZ3 to generate pDrRecG_ΔN99_ as described above. To produce RecG mutant proteins, EcRecG with QPW instead of ^99^FSA^101^ and DrRecG with FSA instead of ^201^QPW^203^, mutagenesis was carried out using fusion PCR techniques. Complementary primer pairs, EcQPW-F/-R and DrFSA-F/-R, were designed to contain the desired mutations in the middle of the primers. To introduce the triple point mutation “QPW” into EcRecG, in the first step, two fragments were amplified using the primer pairs B3652F/EcQPW-R and EcQPW-F/B3652R, respectively. In the second step, the two PCR products were annealed at their overlapping homologous regions and were amplified by the 3652F/R primer pair. The fusion PCR product was cloned into pRADZ3 to generate pEcRecG_QPW_ as described above. The plasmid pDrRecG_FSA_ was constructed using primer pairs DR1916F/DrFSA-R and DrFSA-F/DR1916R. The plasmid constructs were transformed into Δ*recG*. The plasmids were verified by DNA sequencing. Primers used in this study are listed in Supplementary Table S1.

### Construction and Purification of RecG Wedge Domain

A maltose-binding protein (MBP)-RecG-WD fusion was constructed using the pMAL-c2x vector, in which the *E. coli malE* gene encoding MBP was expressed *via* an IPTG-inducible promoter. The WD regions of *recG* genes were amplified using the DrWD-F/-R and EcWD-F/-R primer sets (Supplementary Table S1) by using *D. radiodurans* and *E. coli* genomic DNA as template, respectively. The PCR products were digested with EcoRI and HindIII and cloned into pMAL-c2x for purification of the wild-type WDs, named DrRecG-WD_WT_ and EcRrecG-WD_WT_, respectively. To generate mutant RecG-WDs, DrRecG-WD_FSA_, and EcRecG-WD_QPW_, the WD regions were amplified from plasmids pDrRecG_FSA_ and pEcRecG_QPw_, respectively, and then cloned into pMAL-c2x as described above. The resulting plasmids were transformed into *E. coli* strain BL21 (DE3) for purification of the RecG-WDs fused to MBP. Cells were grown in 500 ml of LB broth at 37°C. IPTG (0.1 mM) was added to the culture when the cells reached an optical density at 600 nm (OD_600_) of 0.5 and were further grown at 16°C overnight. The cells were harvested by centrifugation and resuspended in buffer A (20 mM Tris-HCl, pH 7.5; 0.2 M NaCl; and 1 mM EDTA, pH 8.0). Following sonication on ice, cell debris was removed by centrifugation, and the supernatant was loaded onto a 5 ml amylose column. The column was washed with five column volumes of buffer A. The MBP-RecG-WD fusion protein was eluted with 5 ml of buffer B (buffer A containing 10 mM maltose). Following 10% SDS PAGE, fractions containing the approximately 51-kDa fusion protein were collected and concentrated by ultrafiltration using Amicon® Ultra Filters (Merck Millipore, Darmstadt, Germany). Protein concentrations were determined using the Bradford protein assay with bovine serum albumin as the standard.

### DNA Binding Assay

To create the HJ DNA substrate, four complementary oligonucleotides (oligo 1, 5'-GTCGGATCCTCTAGACAGCTCCATGATCACTGGCACTGGTAGAATTCGGC-3'; oligo 2, 5'-CAACGTCATAGACGATT ACATTGCTACATGGAGCTGTC TAGAGGATCCGA-3'; oligo 3, 5'-TGCCGAATTCTACCAGT GCCAGTGATGGACATCTTTGCCACGTTGACCC-3'; oligo 4, 5'-TGGGTCAACGTGGGCAAGATGTCCTAGCAATGTAATCGTCTATGACGT-3') were synthesized as described previously (McGlynn et al., 2000). The oligonucleotides were denatured for 15 min at 95°C and allowed to anneal at 25°C for 45 min. To examine the DNA binding activity of RecG-WD, an electrophoretic mobility shift assay kit (Invitrogen, Carlsbad, CA, United States) was used. Purified recombinant RecG-WD proteins and 100 nM of HJ DNA substrates were mixed in binding buffer containing 50 mM Tris-HCl (pH 8.0), 5 mM EDTA, 1 mM dithiothreitol, 100 μg/ml bovine serum albumin, and 6% glycerol (v/v) and incubated on ice for 15 min as described previously (McGlynn et al., 1997; Briggs et al., 2005). The DNA binding reaction was terminated by the addition of 2 μl of 2× loading dye. Samples were loaded onto 6% polyacrylamide gels in low-ionic strength buffer at 120 V for 35 min at room temperature, and the electrophoresed DNA-protein complexes were visualized using a ChemiDoc™ Touch imaging system (Bio-Rad, Hercules, CA, United States). Reaction products were quantified using Image Lab Software (Bio-Rad).

### Growth and Survival Assays

A stationary-phase culture that had grown overnight was used as a seed culture. The seed culture was inoculated into 25 ml of TGY broth at 1:100 dilution, and cell growth was monitored at OD_600_ by using a spectrophotometer. For survival studies, cells grown to log phase (OD_600_ ≈ 1.0, corresponding to ~10^8^ CFU/ml) in TGY broth were adjusted to OD_600_ ≈ 0.1 with TGY. After γ-irradiation treatment, cells were serially diluted 10-fold in saline (0.85% NaCl), spotted onto TGY plates, and incubated at 30°C for 3 days to allow colony formation.

### Quantitative Real-Time PCR Assay

A 5 ml culture grown to mid-log phase was harvested by centrifugation. RNA preparation and cDNA synthesis were performed as described previously (Jeong et al., 2016). The quantitative real-time PCR (qRT-PCR) was performed on a CFX Connect real-time PCR system (Bio-Rad) using SYBR Premix Ex Taq (Takara Bio Inc., Otsu, Japan). PCR reactions were performed as follows one cycle of 95°C for 5 min and then 40 cycles of 95°C for 10 s and 60°C for 15 s. The housekeeping gene *dr1343* encoding glyceraldehyde-3-phosphate dehydrogenase was used as an internal control. Primers used in qRT-PCR are denoted with the prefix “RT-” in Supplementary Table S1.

### Pulsed Field Gel Electrophoresis

Irradiated (6 kGy) and unirradiated cells were diluted in 50 ml TGY broth to an OD_600_ of 0.25, and incubated at 30°C. At the indicated times, 1.5, 3, 4.5, 6, and 8 h after re-inoculation, cells (~ 5 × 10^8^ CFU/ml) were harvested and washed with 10 mM phosphate buffer (pH 7.0). The cells were resuspended in 0.125 M EDTA (pH 8.0) and mixed with low-melt agarose (Bio-Rad) to obtain a final concentration of 0.8% agarose. The DNA plugs were incubated overnight at 37°C in 0.05 M EDTA (pH 7.5) containing 1 mg/ml lysozyme, placed in proteinase K solution at 50°C for 16 h, and washed once with 1 × washing buffer containing 1 mM phenylmethylsulfonyl fluoride (PMSF) and then three times with 1× washing buffer. The prepared DNA plugs were digested with 30 U of NotI restriction enzyme overnight at 37°C and then subjected to pulsed field gel electrophoresis (PFGE). DNA fragments were separated on 1% (wt/vol) agarose gels in 0.5 × TBE. Electrophoresis was performed using a CHEF-Mapper apparatus (Bio-Rad) under the following conditions: pulse ramping from 10 to 60 s, angle of 120°, current of 6 V/cm, and 22 h run time at 12°C. Gels were stained with 0.5 × TBE containing 0.5 μg/ml ethidium bromide for 30 min, destained for 10 min in deionized water, and then visualized using a ChemiDoc™ Touch imaging system (Bio-Rad).

## Results

### QPW Residues Are Highly Conserved in the WD of Deinococcal RecG Proteins

**Figure 1:**
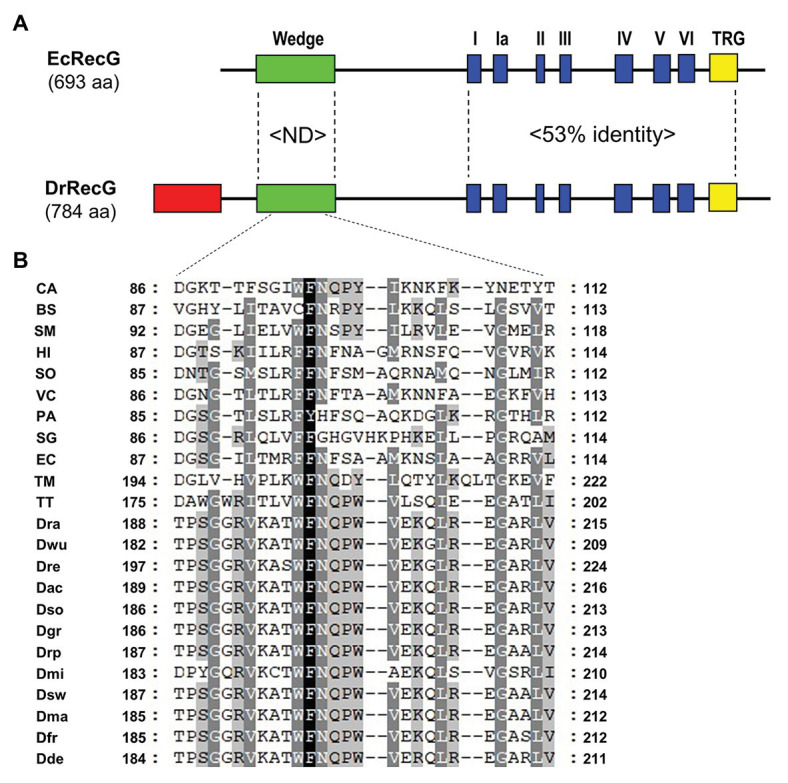
Alignment of *Escherichia coli* RecG (EcRecG) and *Deinococcus radiodurans* RecG (DrRecG). **(A)** Schematic comparison of the domain structures of EcRecG and DrRecG. EcRecG and DrRecG are aligned *via* wedge (green), helicase (blue), and TRG (yellow) motifs. The N-terminal extended region of DrRecG is shown as a red box. The percentages of conservation of amino acid sequences between EcRecG and DrRecG are indicated for wedge domain (ND, not determined) and helicase and TRG motifs (53% identity). **(B)** Multiple alignment of amino acid sequences containing the conserved phenylalanine (F) residue within the wedge domain. The program Genedoc (www.psc.edu/biomed/genedoc) was used to visualize the alignment in quantify mode, which highlights residues most-frequently found in each column of the alignment. Gaps introduced to maximize alignment are indicated by dash. Black and white letters on gray shading represent ≥60 and ≥80% identity, respectively. White letters on black shading represent 100% identity. Wedge domain sequences were obtained from RecGs of *Clostridium acetobutylicum*, CA; *Bacillus subtilis*, BS; *Streptobacillus moniliformis*, SM; *Haemophilus influenza*, HI; *Shewanella oneidensis*, SO; *Vibrio cholera*, VC; *Pseudomonas aeruginosa*, PA; *Streptomyces griseus*, SG; *Escherichia coli*, EC; *Thermatoga maritima*, TM; *Thermus thermophilus*, TT; *D. radiodurans*, Dra; *D. wulumuqiensis*, Dwu; *D. reticulitermitis*, Dre; *D. actinosclerus*, Dac; *D. soli*, Dso; *D. grandis*, Dgr; *D. radiopugnans*, Drp; *D. maricopensis*, Dmi; *D. swuensis*, Dsw; *D. marmoris*, Dma; *D. frigensis*, Dfr; and *D. deserti*, Dde.

In general, RecG proteins largely consist of an N-terminal WD critical to DNA binding, helicase core domains, including seven motifs common to DNA helicases, and the signature RecG TRG motif (Fairman-Williams et al., 2010). Compared to the *E. coli* RecG protein (EcRecG), the *D. radiodurans* RecG protein (DrRecG) has an extended region with 99 amino acids at the N-terminus (Figure 1A). When the C-terminal regions containing the helicase domain and TRG motif are considered, sequence identity between the two proteins approaches 53%, whereas there is no significant similarity in the WD (Figure 1A; Supplementary Figure S1). The crystal structure of RecG from *Thermatoga maritima* (TmRecG) revealed that Phe^204^ and Tyr^208^ in its WD are important for the binding of branched DNA molecules (Singleton et al., 2001). Multiple sequence alignment of the WD showed that the phenylalanine residue was highly conserved, and the tyrosine residue was replaced with another aromatic amino acid, tryptophan, in all deinococcal RecG proteins (Figure 1B; Supplementary Figure S2). Interestingly, glutamine (Q), proline (P), and tryptophan (W) at positions 201, 202, and 203, respectively (*D. radiodurans* numbering) were found in all deinococcal RecGs (Supplementary Figure S2).

### Comparative Modeling of RecG Proteins

**Figure 2:**
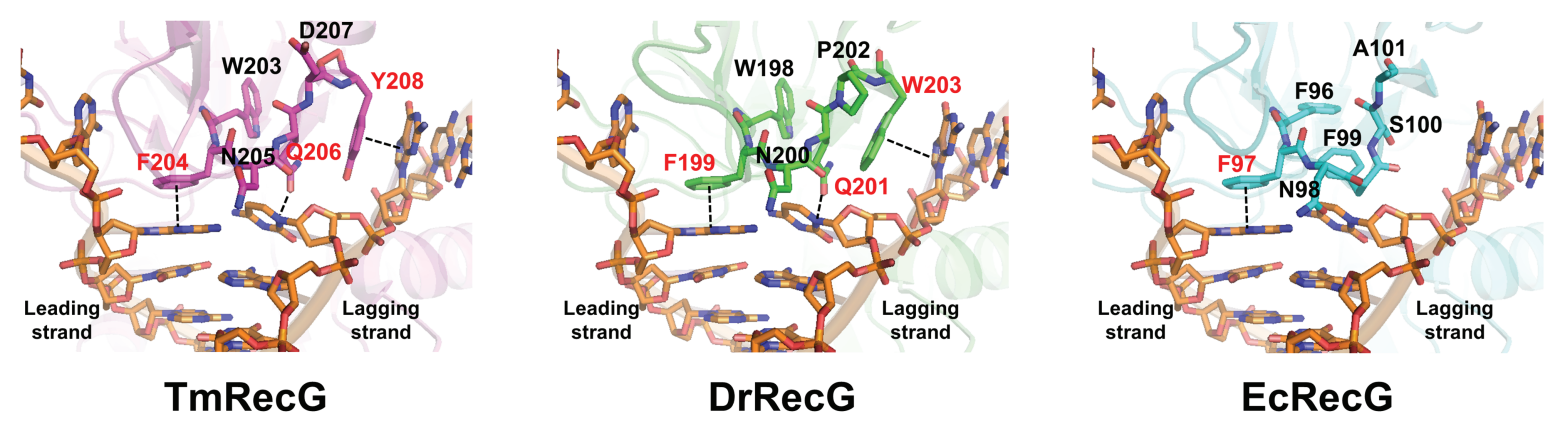
Structural model of RecG wedge domains in complex with a partial replication fork. The modeled wedge domains of RecG from *Thermatoga maritima* (TmRecG), DrRecG, and EcRecG are shown in purple, green, and cyan, respectively. DNA molecules are displayed in an orange stick model. Nitrogen, oxygen, and phosphorous atoms are colored in blue, red, and orange, respectively.

To gain insight into the forked-DNA binding mode of DrRecG, we generated structural models of DrRecG and EcRecG with SWISS-MODEL (Biasini et al., 2014) by using the crystal structure of TmRecG (PDB id: 1gm5) as a template. TmRecG (780 amino acids) shares 36.5 and 32.3% sequence identities with DrRecG (784 amino acids) and EcRecG (693 amino acids), respectively. We predicted structural models for DrRecG comprising of 735 amino acid residues (10–744) and EcRecG comprising of 635 amino acids (6–660), respectively. The modeled structures were validated by using QMEAN and ProSA (Supplementary Figure S3) and were superimposed onto the TmRecG-DNA complex structure. Our analyses suggested that Phe^199^ and Trp^203^ in DrRecG, which are likely to be equivalent to Phe^204^ and Tyr^208^, respectively, in TmRecG, may have roles in the stabilization of the leading and lagging strand templates by base stacking interactions between nucleotide bases and aromatic side chains, whereas Gln^201^ corresponding to Gln^206^ in TmRecG could interact with nucleotide bases by electrostatic interactions, thereby participating in the binding of DrRecG to DNA (Figure 2). In the case of EcRecG, only Phe^97^, corresponding to Phe^204^ in TmRecG, seemed to directly interact with a DNA base (Figure 2). Phe^96^ in EcRecG is also required for interaction with branched DNA molecules because it may have a stabilizing effect similar to that of Trp^203^ in TmRecG, which is partially buried in WD hydrophobic core and preserves WD conformation as a whole (Briggs et al., 2005). Phe^96^ in EcRecG, Trp^203^ in TmRecG, and Trp^198^ in DrRecG, which is equivalent to Trp^203^ in TmRecG, are located in the same position (Figure 2). Taken together, our homology models obtained for DrRecG and EcRecG fit well with the crystal structure of TmRecG. It can thus be assumed that QPW residues may play important roles in the DNA binding of deinococcal RecGs.

### QPW Residues Enhance RecG DNA Binding Activity

**Figure 3:**
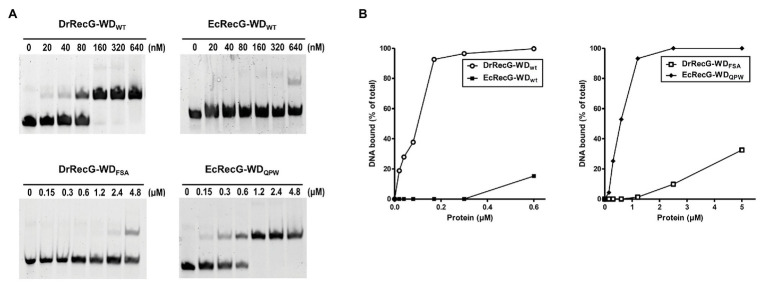
DNA binding activity of RecG wedge domains (RecG-WDs). **(A)** Binding affinities of RecG-WDs as measured in band shift assays with Holiday junction (HJ) substrate. Native RecG-WDs, DrRecG-WD_WT_ and EcRecG-WD_WT_ from *D. radiodurans* and *E. coli*, respectively, and their mutants, DrRecG-WD_FSA_ and EcRecG-WD_QPW_ were used in this assay. Reactions contained 100 nM HJ DNA and native RecG-WDs or mutant RecG-WDs at as shown. **(B)** Quantification of the binding activity of RecG-WDs. The formation of RecG-WD complexes with HJ in **(A)** was quantified and plotted as a function of increasing concentration of RecG-WDs.

The isolated WD can bind to HJ structures, although its affinity is lower than that of full-length RecG (Briggs et al., 2005). To test whether the QPW residues could affect the DNA binding activity of RecG, we cloned partial *recG* gene fragments encoding the WD into the expression vector pMAL-c2x to produce an MBP-RecG-WD fusion protein. The constructs encompassed residues 59–135 of EcRecG, and 158–235 of DrRecG (Supplementary Figure S1). We also constructed mutant versions of RecG-WD by replacing the QPW of DrRecG with FSA of EcRecG corresponding to QPW in DrRecG (Figure 1B), and vice versa. DNA binding assays involving the four different kinds of WDs, EcRecG-WD_WT_, EcRecG-WD_QPW_, DrRecG-WD_WT_, and DrRecG-WD_FSA_, were performed using HJ structures formed by annealing four complementary oligonucleotides, as previously described (McGlynn et al., 2000). As shown in Figure 3, DrRecG-WD_WT_ almost reached a saturation of the total DNA binding at 160 nM protein, whereas EcRecG-WD_WT_ did not even at higher concentrations (to 640 nM). The substitution of QPW with FSA (DrRecG-WD_FSA_) dramatically reduced binding ability, but EcRecG-WD_QPW_ exhibited a significant increase compared to EcRecG-WD_WT_ (Figure 3). These results clearly indicated that the QPW signature motif in deinococcal RecGs plays a role in enhancing the DNA-binding affinity of RecG.

### N-Terminal Extended Region Deletion Does Not Affect DrRecG Activity

**Figure 4:**
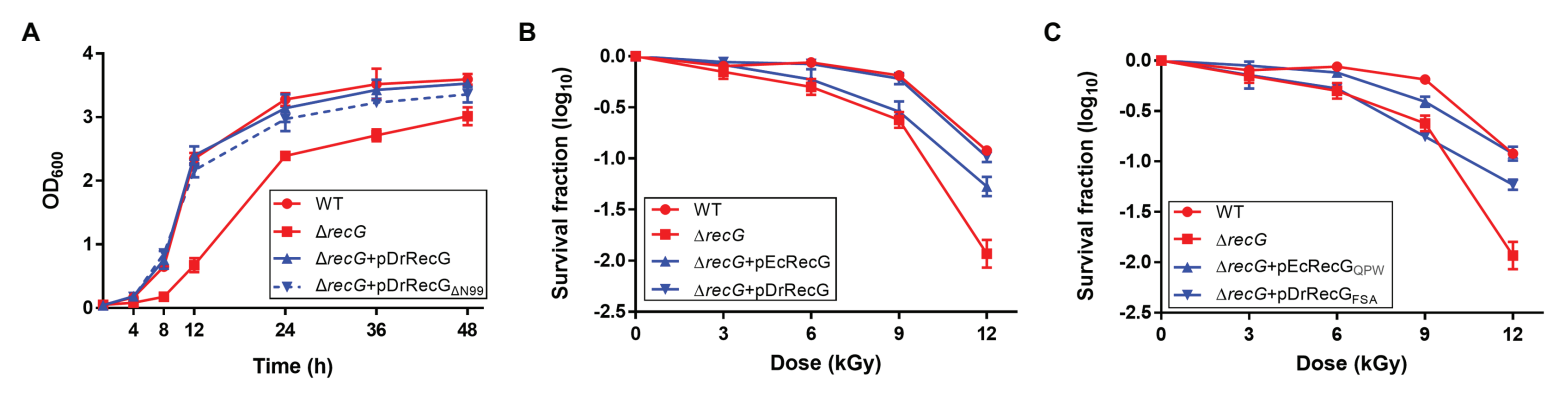
Growth and survival assays of the *D. radiodurans recG* mutant strain (Δ*recG*). **(A)** Growth curves of *D. radiodurans* strains. Optical density (OD_600_) measurements were employed to estimate the growth of *D. radiodurans* R1 (WT), Δ*recG*, and Δ*recG* harboring the plasmids pDrRecG and pDrRecGΔ_N99_, which encode the full-length DrRecG and the N-terminal truncation mutant of DrRecG, respectively. Survival curves for Δ*recG* with pEcRecG and pDrRecG **(B)** and with pEcRecG_QPW_ and pDrRecG_FSA_ **(C)**. Cells grown to log phase were exposed to γ-irradiation and spotted onto TGY plates. The survival fraction was calculated by dividing the colony-forming units (CFUs) of γ-irradiation-treated cells by the CFUs of unirradiated cells. The error bars represent the SD of three independent experiments conducted in duplicate (*n* = 3).

*Deinococus radiodurans* RecG has an extended region, with 99 amino acids in the N-terminus compared to EcRecG (Figure 1A). We constructed plasmid pDrRecG_*Δ*N99_ expressing the N-terminal deletion mutant (residues 100–784) of DrRecG, with Ala^100^ converted to methionine, and transformed it into a DrRecG mutant strain (Δ*recG*). Disruption of *recG* is known to result in *D. radiodurans* cell growth defects (Wu et al., 2009). Thus, we first monitored the growth of the wild-type *D. radiodurans* strain (WT) and Δ*recG* by measuring their OD_600_ values over time. Δ*recG* exhibited delayed growth relative to WT, and expression of the native DrRecG protein (pDrRecG) was able to complement the growth defects. Interestingly, the growth of Δ*recG* harboring pDrRecG_ΔN99_ was comparable to that of Δ*recG* harboring pDrRecG (Figure 4A). In addition, the resistance of Δ*recG* to γ-irradiation was fully restored to the levels of WT by DrRecG_ΔN99_ provided *in trans* (data not shown). These observations indicate that the N-terminal extended region is not involved in the Δ*recG* phenotypes, growth defect, and increased γ-irradiation sensitivity, under the experimental conditions tested here.

### The QPW Motif Increases RecG DNA Repair Capacity

Because the N-terminal extended region was not essential for DrRecG activity (Figure 4A), and EcRecG displays a high degree of sequence conservation with DrRecG in the helicase and TRG motifs (Supplementary Figure S1), we introduced the pEcRecG plasmid carrying the *E. coli recG* gene into Δ*recG* cells and measured cell survival rates following γ-irradiation to determine if EcRecG can functionally replace DrRecG. Compared to WT, Δ*recG* showed approximately 0.25-, 0.5-, and 1-log reductions in survival at 6, 9, and 12 kGy of γ-irradiation, respectively, and these reductions were completely restored by DrRecG. However, EcRecG was able to partially restore the γ-irradiation-sensitive phenotype of Δ*recG* only at 12 kGy of γ-irradiation (Figure 4B). To test the functional role of the QPW residues, we constructed pEcRecG_QPW_ encoding an EcRecG mutant in which ^99^FSA^101^ was substituted with QPW, and pDrRecG_FSA_ encoding a DrRecG mutant in which ^201^QPW^203^ was substituted with FSA. The resulting plasmids were transformed into Δ*recG* cells, which were then exposed to γ-irradiation. There was no significant difference in *recG* mRNA levels between Δ*recG* harboring pDrRecG and pDrRecG_FSA_ (Supplementary Figure S4), but resistance to γ-irradiation was not recovered by DrRecG_FSA_ (Figure 4C). In contrast, EcRecG_QPW_ restored the Δ*recG* survival in a similar way as DrRecG, although the restoration levels were somewhat different at 9 kGy (Figures 4B,C). These results showed that substituting the three amino acids in the WD between EcRecG and DrRecG is sufficient to exchange their abilities to complement the loss-of-function phenotype of Δ*recG* after γ-irradiation.

**Figure 5:**
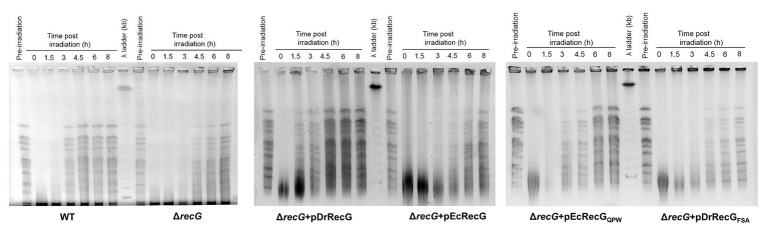
DNA repair in Δ*recG* and Δ*recG* cells harboring plasmids encoding the native (pDrRecG and pEcRecG) and mutant (pDrRecG_FSA_ and pEcRecG_QPW_) RecG proteins. Exponentially growing cells were exposed to 6 kGy of γ-irradiation and then recovered in TGY liquid media. At the indicated times (0–8 h), samples were removed, and genomic DNA was isolated. The amount of DNA double-strand breaks (DSBs) was analyzed by pulsed-field gel electrophoresis (PFGE). Pre-irradiation indicates unirradiated cells.

To investigate the role of DrRecG in DSB, the DNA repair kinetics of cells subjected to γ-irradiation were examined using PFGE. DNA fragmented by γ-irradiation was reconstructed within 3 h in WT, whereas Δ*recG* was able to compete the repair of shattered genomes 4.5 h after γ-irradiation (Figure 5). Δ*recG* harboring pDrRecG and pEcRecG showed repair kinetics similar to those observed in WT and Δ*recG*, respectively. Compared to Δ*recG* with pEcRecG_QPW_, the process of genome reassembly was delayed by 1.5 h in Δ*recG* with pDrRecG_FSA_ (Figure 5). The similar patterns of results shown in survival assays and PFGE analysis, which are caused by the reciprocal swapping of the three amino acid motifs between DrRecG and EcRecG, strongly suggest that the QPW residues enhance DNA-binding activity, thereby leading to efficient DNA repair in *D. radiodurans*.

## Discussion

Analysis of the genome sequence of *D. radiodurans* identifies the typical complement of prokaryotic DNA repair proteins, suggesting the possibility that *D. radiodurans* uses the same DNA-repair strategies as other prokaryotes, but it does so in a manner that is somehow much more effective than that observed in other species to retain the extraordinary tolerance to DNA damage (Battista et al., 1999; White et al., 1999). A series of biochemical and structural analyses of *D. radiodurans* proteins involved in DNA repair systems have revealed unusual features distinguishing them from their counterparts in other prokaryotic species. Uracil-DNA glycosylase (UNG) removes uracil from DNA molecules, formed as a result of cytosine deamination, and represents part of the base-excision repair pathway. The uracil-DNA *N*-glycosylase DrUNG (DR_0689), the main UNG in *D. radiodurans*, possesses a much higher catalytic efficiency than human UNG, which is attributed to the high substrate affinity caused by an increased number of positively charged residues close to the DNA binding site (Pedersen et al., 2015). Mismatch-specific UNG (MUG) also removes uracil from DNA. The overall structure of DrMUG is similar to that of EcMUG, but DrMUG possesses a novel catalytic residue (Asp-93) that can provide DrMUG with broad substrate specificity (Moe et al., 2006). The novel catalytic residue identified in DrMUG is conserved in other deinococcal MUG proteins (Lim et al., 2019). The *D. radiodurans* X family DNA polymerase (DrPolX), which not only has polymerase activity, but also exerts strong Mn^2+^-dependent 3'→5' exonuclease activity affects DSB repair efficiency in *D. radiodurans* (Blasius et al., 2006). At the active site of the polymerase catalytic domain, the “DXD” motif conserved in almost all pol X-family members is replaced by an “AAE” motif in *D. radiodurans* (Leulliot et al., 2009; Bienstock et al., 2014). RecA is a critical enzyme in HR for DSB repair. Unlike canonical RecAs, DrRecA first forms a filament on dsDNA and then takes up a homologous single strand to initiate DNA strand exchange, which is the exact inverse of the major pathway seen with other proteins of the RecA family (Kim and Cox, 2002). Although the overall fold of DrRecA is similar to EcRecA, there are a few key amino acid changes in the DrRecA C-terminal domain that interacts with dsDNA. One of the key differences, Phe^303^ in DrRecA, equivalent to Trp^290^ in EcRecA, would seem to be a likely candidate in dictating a possible specificity for binding to dsDNA (Rajan and Bell, 2004). It has also been observed that a single amino acid mutation at the C-terminus of EcRecA alters its activity. The RecA variants found in radioresistant *E. coli* strain CB2000 have mutations at residue 276 (D276A and D276N), which increase the rates of filament nucleation on DNA, and promote DNA strand exchange more efficiently than wild-type RecA proteins (Piechura et al., 2015). These studies imply that DNA repair proteins with altered substrate specificity and enhanced catalytic activity, which might be attributed to unique amino acid residues, likely contribute to the improved DNA repair capacity of *D. radiodurans* to survive DNA damage caused by γ-irradiation. In this aspect, the highly conserved QPW residues in the WD can be assumed to affect the binding activity of deinococcal RecGs.

The WD found in the N-terminal region of RecG is a DNA-binding domain that has an oligonucleotide/oligosaccharide binding-fold (OB-fold) motif ranging between 70 and 150 amino acids in length (Theobald et al., 2003). The phenylalanine residue, which is critical for RecG binding of branched DNA molecules, is conserved in the WDs (Briggs et al., 2005; Figure 1B). This residue is regarded to stabilize the orphan base of the leading strand template by base stacking, effectively capping the parental duplex (Singleton et al., 2001; Figure 2). In *Mycobacterium tuberculosis* RecG, substitution of Phe^99^ corresponding to Phe^204^ in TmRecG to Ala was seen to significantly lower the DNA binding activity compared to that of wild-type RecG protein (Zegeye et al., 2014). In this study, protein structure modeling analysis showed that two other amino acids, Gln^206^ and Tyr^208^ in TmRecG and Gln^201^ and Trp^203^ in DrRecG, interacted with the nucleotide bases of the leading and lagging forked-DNA strands, together with the key residues, Phe^204^ in TmRecG, and Phe^199^ in DrRecG, respectively (Figure 2). The aromatic side-chain of Tyr participates in DNA binding of the WD through hydrophobic interactions such as base stacking (Singleton et al., 2001). Tyr was not observed in DrRecG, but it was substituted with an equivalent aromatic residue, Trp (Figure 2), suggesting that Trp^203^ may play a similar role in the stabilization of the lagging DNA strand duplex. Of the 79 deinococcal RecGs analyzed in this study, only two species *Deinococcus ruber* and *Deinococcus aquiradiocola*, have QAW instead of the signature QPW residues (Supplementary Figure S2). However, it is noteworthy that Q and W are strictly conserved. EcRecG does not have a signature motif with an aromatic residue in the corresponding position (^99^FSA^101^ in EcRecG). Comparisons of the DrRecG and EcRecG models suggested that the conserved QPW residues in the WD confer greater RecG-DNA interaction compared to FSA because of the additional charge interactions and base packing through Gln and Trp, respectively (Figure 2; Supplementary Figure S5). Indeed, DrRecG-WD_WT_ and EcRecG-WD_QPW_ exhibited enhanced affinities for HJ compared to EcRecG-WD_WT_ and DrRecG-WD_FSA_ (Figure 3). Full-length DrRecG and EcRecG_QPW_ restored resistance to γ-irradiation and DNA repair capacity of Δ*recG* to that of WT (Figures 4, 5).

Also noteworthy is the N-terminal extended region of DrRecG (Figure 1A). This feature is shared with RecG proteins from thermophilic bacteria, *Thermotoga maritima* and *Aquifex aeolicus*, and a number of cyanobacteria, and is longer than in many other RecG proteins such as EcRecG (Wen et al., 2005). RecG from the extreme thermophile *A. aeolicus* unwinds DNA well at high temperature (60°C), whereas the N-terminal extension present in this protein is dispensable for activity and thermo-stability (Wen et al., 2005). In *D. radiodurans*, the N-/C-terminal extension (or variation) is found not only in RecG, but also in other helicases, including RecD2 and RecQ. DrRecD2 contains an additional N-terminal region of approximately 200 amino acids, which is longer than the corresponding feature in *E. coli* RecD protein (RecD1; Montague et al., 2009). In contrast to most other RecQ proteins composed of the helicase, RecQ-C-terminal (RQC), and helicase-and-RNaseD-like-C terminal (HRDC) domains, DrRecQ contains three tandem HRDC domains in the C-terminus (Killoran and Keck, 2006). Full-length DrRecD2 restored the H_2_O_2_-resistant phenotype of *recD2* mutants, whereas the N-terminal truncation mutant of DrRecD2 could not, suggesting that the N-terminal domain is necessary for the role of DrRecD2 in antioxidant pathways (Zhou et al., 2007). The three tandem HRDC domains increase the efficiency of DNA unwinding and ATPase activities of DrRecQ (Huang et al., 2007), and could work together with other functional motifs to enhance DrRecQ interactions with DNA (Liu et al., 2013). Considering that Δ*recG* cells are sensitive to γ-irradiation and H_2_O_2_ (Wu et al., 2009; Figure 4), and DrRecG is also involved in catalase gene *katE1* regulation (Jeong et al., 2016), further research is needed to determine the role of the additional DrRecG N-terminal region, although it seems not to be implicated in DNA repair under the experimental conditions tested in this study.

RecG provides a more general defense against pathological DNA replication, e.g., the rescue of stalled or damaged replication forks, and is associated with a number of additional cellular processes (Rudolph et al., 2010; Azeroglu et al., 2016; Lloyd and Rudolph, 2016; Romero et al., 2020). Recently, it has become increasingly clear that RecG is an enzyme responsible for regression of stalled DNA replication forks that can be induced by various types of lesions, including single-strand breaks and DSB. RecG is directly loaded onto the forks by binding to ssDNA-binding protein (SSB) and reverses the stalled forks, leading to the formation of HJ-type structures. PriA helicase then targets the repaired fork to reload and assemble a DNA replication machinery (the replisome complex), enabling DNA replication to restart (Lloyd and Rudolph, 2016; Bianco and Lyubchenko, 2017). During HR for DSB repair, RecG is proposed to re-model branched intermediates of recombination to direct the correct binding of PriA and subsequent DNA synthesis (Azeroglu et al., 2016). These studies indicate that SSB-mediated RecG loading onto DNA plays an important role in facilitating stalled replication fork rescue. *D. radiodurans* SSB (DrSSB) is different from that of the prototype *E. coli* SSB (EcSSB), in that DrSSB (301 amino acids) contains two OB folds per monomer, and functions as homodimers, in contrast to the homotetrameric EcSSB (178 amino acids), with each monomer encoding a single OB fold (Bernstein et al., 2004). In *E. coli*, SSB-RecG interactions occur *via* the WD of RecG and the PXXP motifs within the SSB linker domain. In particular, the SSB binding site on RecG overlaps the residues of the binding site for the leading strand arm of the fork in RecG (Ding et al., 2020). Thus, it is likely that these different features do not enable DrSSB to deliver EcRecG to the fork with an efficiency equal to that of DrRecG, which may explain why EcRecG does not confer resistance to Δ*recG* (Figure 4B). EcRecG also partially complements *Helicobacter pylori recG* mutants but not to the same extent as the *H. pylori* RecG protein, suggesting that the host context appears to be critical in defining the function of RecG (Kang et al., 2004). In this aspect, the QPW residues found in the WD of DrRecG might be interpreted as the result of evolutionary adaptation to cooperate with the unique DrSSB. *D. radiodurans* is most closely related to *Thermus thermophilus*, and the two genera *Deinococcus* and *Thermus* belong to a distinct bacterial clade called the *Deinococcus*-*Thermus* group (Omelchenko et al., 2005). Regarding RecG and SSB, they share common features. *Thermus* RecG proteins have either QPW or QTW (Supplementary Figure S6), and the dimeric SSBs like DrSSB are discovered in the *Deinococcus*-*Thermus* group (Zhang et al., 2014), supporting that RecG and SSB might be evolutionarily related. *T. thermophilus* is a thermophile, which is relatively sensitive to IR, whereas *D. radiodurans* is a mesophile, which is highly IR-resistant (Omelchenko et al., 2005). However, considering that the mechanisms employed by thermophiles to overcome DNA damage by high temperature may also be employed in repair of damage caused by IR (Ranawat and Rawat, 2017), the coevolution between RecG and SSB, which likely occurred in the *Deinococcus*-*Thermus* group, may contribute to the enhanced abilities of the *Deinococcus* and *Thermus* lineages to survive different kinds of environmental stresses. Further research is warranted to investigate the effects of QPW on RecG-SSB interactions.

## Data Availability Statement

The original contributions presented in the study are included in the article/Supplementary Material, further inquiries can be directed to the corresponding author.

## Author Contributions

S-WJ performed the experiments and wrote a first draft. M-KK performed the comparative protein structure modeling and revised the first draft. LZ and S-KY constructed the plasmids and performed the survival assays. M-KK, J-HJ, and H-ML guided the experiments and interpreted the results. SL conceived the study and was in charge of overall direction and planning. All authors reviewed and edited the manuscript, and approved the final version.

### Conflict of Interest

The authors declare that the research was conducted in the absence of any commercial or financial relationships that could be construed as a potential conflict of interest.
