# Supplementary material for: Effects of Conserved Wedge Domain Residues on DNA Binding Activity of Deinococcus radiodurans RecG Helicase
Source: Front Genet. 2021 Feb 4;12:634615. doi: 10.3389/fgene.2021.634615 (PMC7889586; doi:10.3389/fgene.2021.634615)
Supplement: Supplementary file 1 [file Data_Sheet_1.pdf]

Supplementary Table S1. Primers used in this study

Primer	Sequence (5’ to 3’)
DR1916F	ttaactagtatgagcgaaatggcgacggtg
DR1916R	ttaggatcctcaaatcacttcgcggtacgc
DR1916-ΔNF	taaactagtatgcccggtgagcgcctgccgc
DR1916-ΔNR	aatggatccctagcggcaaaatggcctgtca
DrFSA-F	gtcaaggcgacgtggttcaacttcagcgcggtcgagaagcagctgcgcgaggcgag
DrFSA-R	ctcgcgcagctgcttctcgaccgcgctgaagttgaaccacgtcgccttgac
B3652F	ttaactagtatgaaaggtcgcctgttagat
B3652R	ttaggatccttacgcattcgagtaacgttc
EcQPW-F	atgcgctttttcaatcaaccctgggcaatgaaaaatagcctg
EcQPW-R	caggctatttttcattgcccagggttgattgaaaaagcgcat
DrWD-F	tagaattcggccagaaggtgaccgtg
DrWD-R	taaagcttttacttgtccaccgtttccat
EcWD-F	taggattcggcgtttatgccacggtg
EcWD-R	taaagcttcggcgtttatgccacggtg
RT-DR1916F	gcggcgaggacatcaacaag
RT-DR1916R	gcgcatgtcgtcggtgattt
RT-DR1343F	ccgccaaccaccacatcatc
RT-DR1343R	atggccttctcgatgccgaa

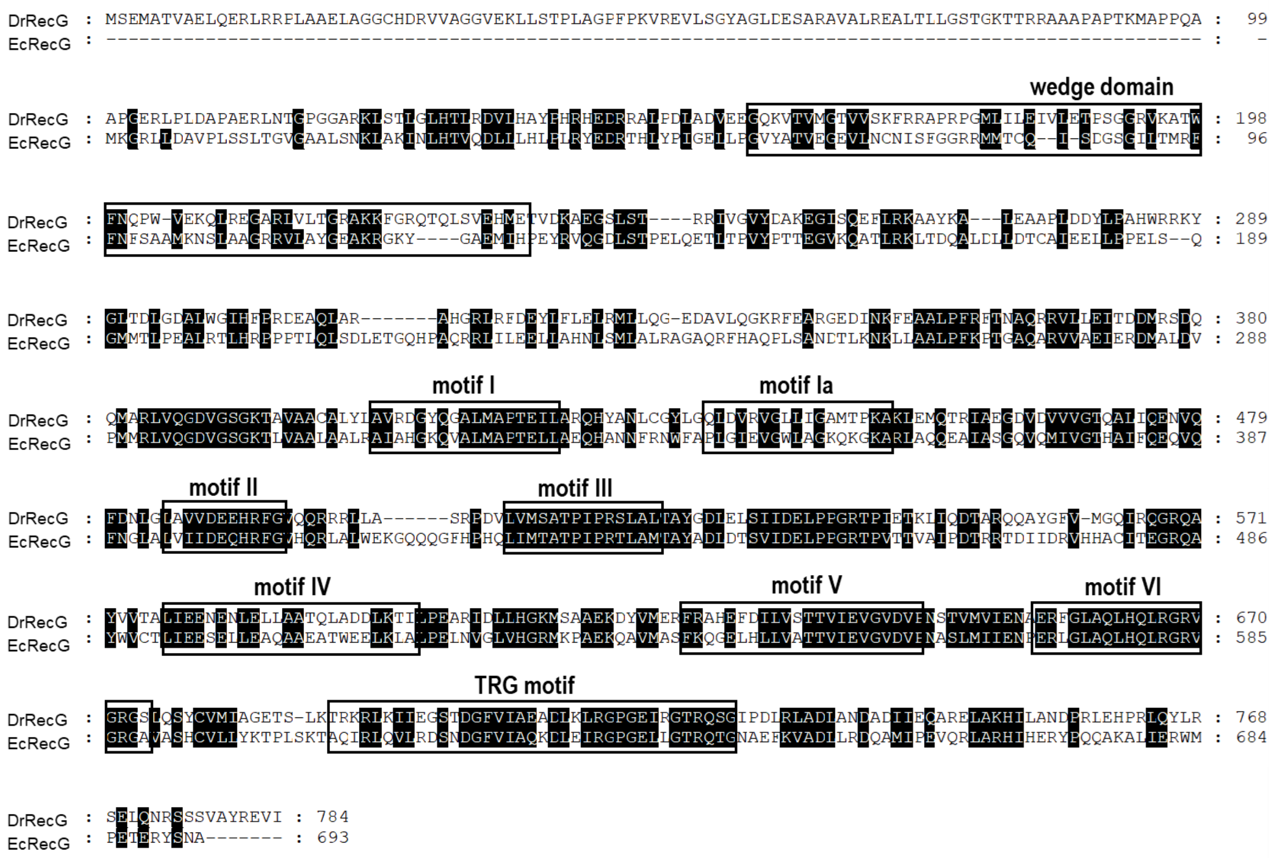

Supplementary Figure S1. Alignment of amino acid sequences of the *D. radiodurans* R1 (DrRecG) and *E. coli* MG1655 RecG (EcRecG) proteins. Protein sequences were aligned using ClustalW. The locations of wedge domain, helicase motifs (I to VI), and TRG (translocation in RecG) motifs are indicated with boxes.

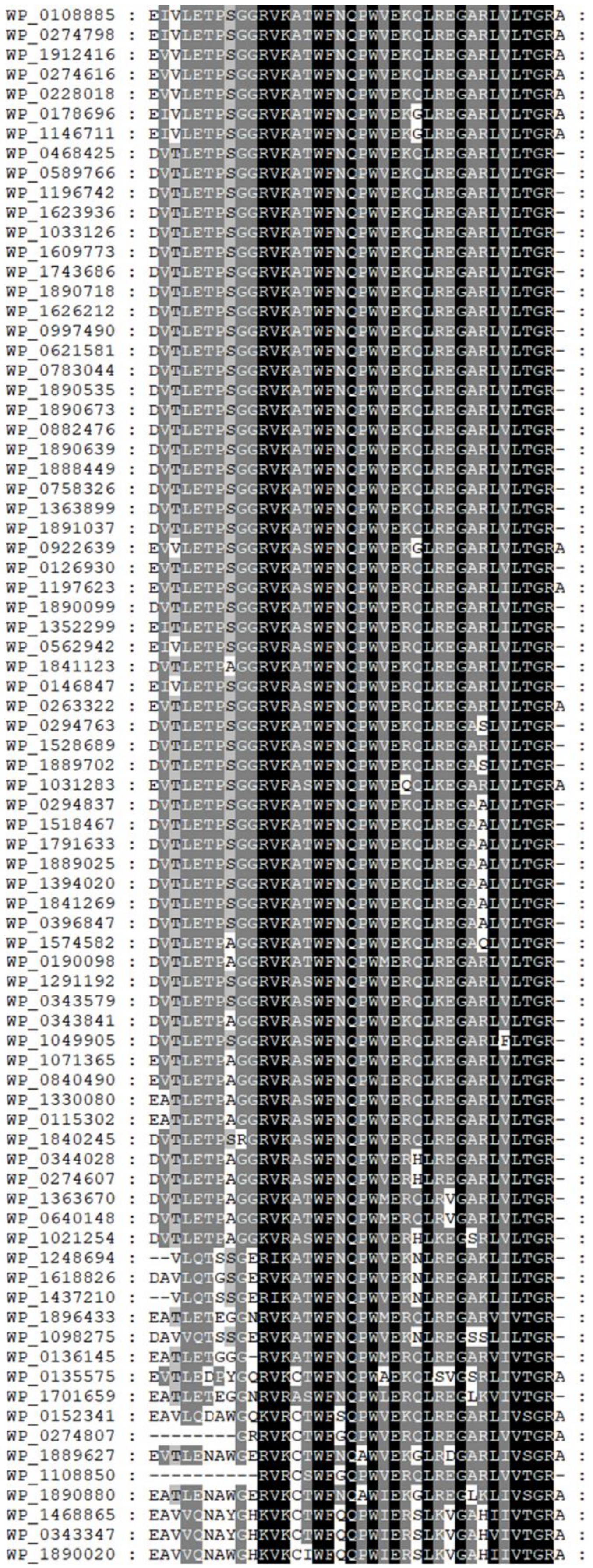

Supplementary Figure S2. Multiple alignment of amino acid sequences of 79 deinococcal RecGs, which contains OPW residues in the wedge domains. Protein sequences obtained from the NCBI database are distinguished by the WP protein accession number. Black and white letters on gray shading represent ≥ 60% and ≥ 80% identity, respectively. White letters on black shading represent 100% identity.

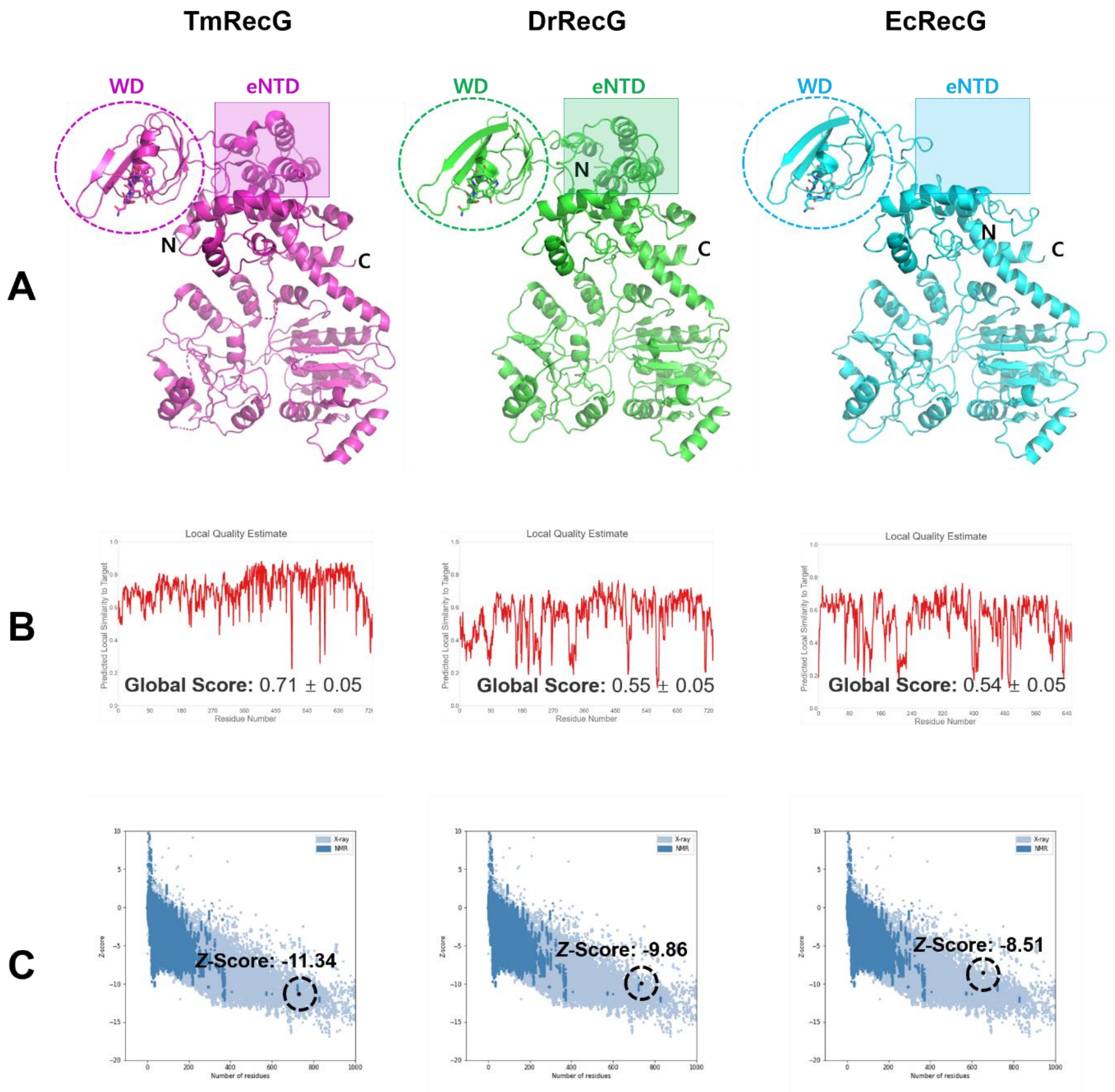

Supplementary Figure S3. (A) Full views of the crystal structure of TmRecG and modelled structures of DrRecG and EcRecG. Wedge domains (WD) are marked with dotted ellipses and extended N-terminal regions (eNTD) are highlighted with filled squares. N and C denote the N- and C-terminal ends, respectively. **(B)** Diagrams of the local quality estimate by QMEAN for the crystal structure of TmRecG and modelled structures of DrRecG and EcRecG. Global score with the standard deviation is shown in each diagram. **(C)** Overall model quality measured by Z-score versus number of residues using ProSA. The position of each RecG structure is marked as black dot and highlighted with dotted black circle. Z-score of each structure is also presented.

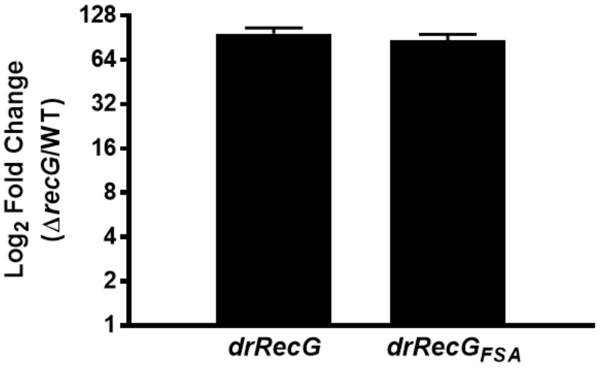

Supplementary Figure S4. qRT-PCR assay of *recG* expression. The *drRecG* and *drRecG$_{FSA}$* mRNA levels were measured in Δ*recG* harboring pDrRecG and pDrRecG$_{FSA}$, respectively. The relative expression values were determined by dividing the mRNA levels from Δ*recG* harboring the plasmids by the mRNA levels from WT. The expression levels of the target genes were normalized to the *dr1343* gene. The means ± standard deviations of three independent experiments performed in duplicate are shown.

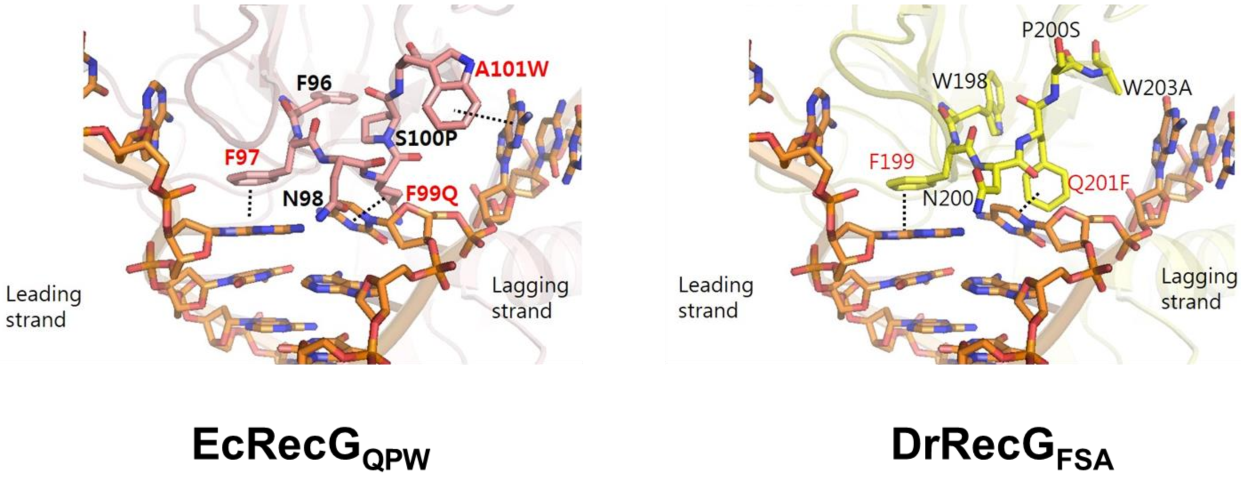

Supplementary Figure S5. Structural model of RecG wedge domains in complex with a partial replication fork. The modeled wedge domains of EcRecG$_{QPW}$ and DrRecG$_{FSA}$ are shown in salmon and yellow, respectively. DNA molecules are displayed in an orange stick model. Nitrogen, oxygen, and phosphorous atoms are colored in blue, red, and orange, respectively.

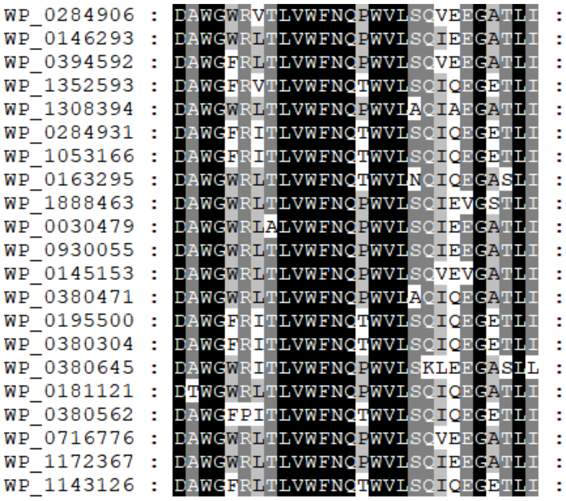

Supplementary Figure S6. Multiple alignment of amino acid sequences of 21 *Thermus* RecGs, which contains either OPW or QTW residues in the wedge domains. Protein sequences obtained from the NCBI database are distinguished by the WP protein accession number. Black and white letters on gray shading represent ≥ 60% and ≥ 80% identity, respectively. White letters on black shading represent 100% identity.
